# Mesenchymal stromal cells-derived extracellular vesicles reprogramme macrophages in ARDS models through the miR-181a-5p-PTEN-pSTAT5-SOCS1 axis

**DOI:** 10.1136/thoraxjnl-2021-218194

**Published:** 2022-08-10

**Authors:** Yue Su, Johnatas Dutra Silva, Declan Doherty, David A Simpson, Daniel J Weiss, Sara Rolandsson-Enes, Daniel F McAuley, Cecilia M O'Kane, Derek P Brazil, Anna D Krasnodembskaya

**Affiliations:** 1 Wellcome-Wolfson Institute for Experimental Medicine, School of Medicine, Dentistry and Biomedical Sciences, Queen's University Belfast, Belfast, UK; 2 Department of Medicine, University of Vermont, Burlington, Vermont, USA; 3 Department of Experimental Medical Science, Faculty of Medicine, Lund University, Lund, Sweden

**Keywords:** ARDS, macrophage biology

## Abstract

**Rationale:**

A better understanding of the mechanism of action of mesenchymal stromal cells (MSCs) and their extracellular vesicles (EVs) is needed to support their use as novel therapies for acute respiratory distress syndrome (ARDS). Macrophages are important mediators of ARDS inflammatory response. Suppressor of cytokine signalling (SOCS) proteins are key regulators of the macrophage phenotype switch. We therefore investigated whether SOCS proteins are involved in mediation of the MSC effect on human macrophage reprogramming.

**Methods:**

Human monocyte-derived macrophages (MDMs) were stimulated with lipopolysaccharide (LPS) or plasma samples from patients with ARDS (these samples were previously classified into hypo-inflammatory and hyper-inflammatory phenotype) and treated with MSC conditioned medium (CM) or EVs. Protein expression was measured by Western blot. EV micro RNA (miRNA) content was determined by miRNA sequencing. In vivo: LPS-injured C57BL/6 mice were given EVs isolated from MSCs in which miR-181a had been silenced by miRNA inhibitor or overexpressed using miRNA mimic.

**Results:**

EVs were the key component of MSC CM responsible for anti-inflammatory modulation of human macrophages. EVs significantly reduced secretion of tumour necrosis factor-α and interleukin-8 by LPS-stimulated or ARDS plasma-stimulated MDMs and this was dependent on SOCS1. Transfer of miR-181a in EVs downregulated phosphatase and tensin homolog (PTEN) and subsequently activated phosphorylated signal transducer and activator of transcription 5 (pSTAT5) leading to upregulation of SOCS1 in macrophages. In vivo, EVs alleviated lung injury and upregulated pSTAT5 and SOCS1 expression in alveolar macrophages in a miR181-dependent manner. Overexpression of miR-181a in MSCs significantly enhanced therapeutic efficacy of EVs in this model.

**Conclusion:**

miR-181a-PTEN-pSTAT5-SOCS1 axis is a novel pathway responsible for immunomodulatory effect of MSC EVs in ARDS.

**WHAT IS ALREADY KNOWN ON THIS TOPIC**

- Although it is widely accepted that macrophages modulation is a major mechanism of mesenchymal stromal cells (MSC) actions in inflammatory conditions, specific signalling pathways activated by MSCs in primary human macrophages remain largely unknown.

**WHAT THIS STUDY ADDS**

- MSC extracellular vesicles contain miR-181a-5p. This micro RNA reduces expression of phosphatase and tensin homolog in macrophages, leading to signal transducers and activators of transcription 5 phosphorylation and induction of the inhibitory regulator, suppressor of cytokine signalling 1 (SOCS1) protein, with a consequent reduction in macrophage inflammatory cytokine secretion. Upregulation of SOCS1 in macrophages is critical for the immunomodulatory effects of MSCs in clinically relevant models of acute respiratory distress syndrome (ARDS).

**HOW THIS STUDY MIGHT AFFECT RESEARCH, PRACTICE AND/OR POLICY**

- These findings reveal a new mechanism of action of MSCs and MSC extracellular vesicles in ARDS and identify a novel signalling pathway which could be exploited therapeutically for the treatment of ARDS.

## Introduction

Acute respiratory distress syndrome (ARDS) and sepsis are the biggest cause of mortality in critically ill patients with no specific pharmacological treatment.^1 2^ ARDS is a syndrome where heterogeneity of the underlying pathophysiological mechanisms presents a significant obstacle for translational research.^3 4^ Based on the circulating inflammatory biomarkers, patients with ARDS can be classified into subpopulations characterised by different patterns of inflammatory response.^5^ Using the method of latent class analysis, two broad biological phenotypes (hyper-inflammatory and hypo-inflammatory) were identified retrospectively in two large randomised clinical trials and in one observational study. The phenotypes had different clinical outcomes and responded differently to pharmacological and therapeutic interventions.^6–9^ Therefore, it is important to further investigate the underlying biological mechanisms responsible for differences in the inflammatory responses between ARDS phenotypes and where possible, consider the investigation of potential new therapeutics separately for each phenotype.

Mesenchymal stromal cells (MSCs) are being actively investigated as a cell-based therapy for ARDS.^10 11^ Clinical trials using MSCs in ARDS (including COVID-19-induced ARDS) are ongoing. Macrophages are key innate immune cells responsible for orchestrating inflammatory responses and are critical in driving inflammation and injury in ARDS.^12 13^ Our group and other investigators have demonstrated that macrophages are important cellular mediators of MSC immunomodulatory and anti-microbial effects in a range of inflammatory conditions including pneumonia and sepsis.^14 15^ Importantly, we have shown that depletion of alveolar macrophages results in the abrogation of the protective effects elicited by MSCs in the in vivo *Escherichia coli* pneumonia model of lung injury.^16^ Furthermore, we have demonstrated that in pre-clinical models of ARDS, MSCs reprogramme both human and murine macrophages towards M2-like, anti-inflammatory phenotype with enhanced phagocytic activity, an effect, that is, at least partially mediated by mitochondrial transfer resulting in macrophage metabolic reprogramming.^16 17^ One of the important questions, which still needs to be addressed is: how do MSCs modulate intracellular signalling in their target cells?

It is widely accepted that therapeutic effects of MSCs are mediated largely by their secretome. Importantly, MSC-derived extracellular vesicles can recapitulate many effects of MSCs themselves and are being developed as a cell-free therapeutic for multiple conditions including ARDS.^18 19^ MSC extracellular vesicles (EVs) are enriched in regulatory micro RNA (miRNA) content which has a potential for modulation of functional properties of recipient cells,^20^ however, the functional importance of individual miRNAs within the EV cargo remains largely unknown.

Suppressor of cytokine signalling (SOCS) proteins are inducible feedback inhibitors of the Janus kinase/signal transducers and activators of transcription (JAK/STAT) signalling pathway. The mammalian SOCS protein family consists of at least eight members, SOCS1–7 and CIS (cytokine‐inducible Src homology 2 protein).^21^ Data from transgenic animal studies suggest that these regulatory proteins play a key role in macrophage polarisation.^22^ Of particular interest is SOCS1, which has been shown to be a multifunctional inhibitor of the inflammatory response and capable of preventing activation of pathogen recognition receptors, cytokine receptors and receptors for growth factors.^23^ It has been demonstrated that SOCS1 expression is critical for the control of M2 polarisation both in vitro and in vivo.^24^ Additionally, mice containing a myeloid-specific deletion in SOCS1 have been reported to be more susceptible to sepsis.^25^ However, the role of SOCS1 protein in regulation of human macrophages in the context of ARDS and its contribution to the MSC therapeutic effect is currently unknown.

In the present study, we aimed to investigate the role of SOCS1 in the modulation of human macrophages by MSCs and decipher the mechanisms underpinning this effect.

We hypothesised that SOCS1 is crucial for the reprogramming of human macrophages and reduction of inflammation by MSCs in the ARDS environment and that MSCs modulate SOCS1 expression via the transfer of miRNAs in extracellular vesicles. We also hypothesised that therapeutic efficacy of MSC EVs may be enhanced by manipulating EV miRNA expression. Some of the results of these studies were previously reported in the form of abstracts.^26 27^

## Materials and methods

Detailed methods are described in the online supplemental document.

10.1136/thoraxjnl-2021-218194.supp1 Supplementary data

### Cell culture

Human bone marrow-derived MSCs were acquired from the Institute for Regenerative Medicine at Texas A&M University, (Temple, Texas, USA) and the American Type Culture Collection (LGC Standards UK). These cells fulfil all requirements set by the International Society of Cellular Therapy for defining MSCs.^28^ Human monocyte-derived macrophages (MDMs) were generated using granulocyte macrophage colony-stimulating factor (GM-CSF) (R&D systems, UK) differentiation (10 ng/mL for 7 days) of monocytes from buffy coats obtained from the Northern Ireland Blood Transfusion Service.

### Generation of MSC conditioned medium and extraction of MSC-derived EVs

MSC conditioned medium (CM) was generated from MSCs cultured in RPMI-1640 with 1% fetal bovine serum (FBS) for 24 hours. For MSC EV isolation, MSCs were cultured in serum-free α-MEM-medium for 48 hours before EV extraction using ultracentrifugation as previously described.^29^ EVs were resuspended in phosphate-buffered saline (PBS) and characterised according to the International Society for Extracellular Vesicles^30^ guidelines (online supplemental figure 1).

10.1136/thoraxjnl-2021-218194.supp2 Supplementary data

### Co-culture of MDMs with MSC CM and MSC EVs

MDMs were treated with MSC CM or EVs in the presence of *E. coli* lipopolysaccharide (LPS) O111:B4 (Millipore) (10 ng/mL) or 10% plasma or 30% bronchoalveolar lavage fluid (BALF). Plasma samples used in the study were from patients recruited to HARP-2 study.^31^ These samples were previously classified into two phenotypes based on concentrations of plasma inflammatory biomarkers.^7^ Ten plasma samples representative of each phenotype were pooled and diluted in 1% complete medium to final concentration of 10% before use, plasma from healthy volunteers was used as a control. BALF samples were from HARP study,^32^ nine BALF samples were pooled to generate a stock and the pooled sample was then diluted to 30% in RPMI 1% FBS+Penicillin/Streptomycin (PS) before stimulation. Only baseline samples obtained prior to intervention were used for experiments. Ethical approval for use of patient samples for research was granted by the Office for Research Ethics Committees Northern Ireland.

### Small RNA sequencing of EVs

MSCs were exposed to pooled ARDS BALF (30%) for 24 hours,^32^ cell supernatants were collected for EV isolation, cells washed and RNA isolated. EVs were isolated from cell supernatants and BALF by ultracentrifugation. RNA was extracted using ‘miRNeasy’ Kit, (Qiagen). RNA integrity was assessed on Qubit RNA HS Assay Kit (Invitrogen). Small RNAs were converted to complementary DNA libraries using the NEXTFLEX Small RNA-Seq Kit V.3 (PerkinElmer). Quality control of the libraries and sequencing was performed by the Genomics Core Technology Unit at Queens University Belfast on a NextSeq 550 System (Illumina). FASTq files were uploaded onto CLC Genomics Workbench and analysed using the small RNA pipeline analysis tool (Qiagen Digital Insights). This tool was used for trimming of sequencing reads, counting, annotation of the results using miRBase V.21 and differential expression analysis.

### In vivo LPS-induced lung injury model

All animal experiments were approved by Animal Welfare Ethical Review Body of Queen’s University Belfast, in accordance with UK Animals (Scientific Procedures) Act 1986. C57BL/6 male mice (8–12 w.o., Envigo RMS (UK) Station Road Blackthorn Bicester Oxon) were used. Mice were anaesthetised by xylazine/ketamine (0.25 mg/kg and 0.025 mg/kg, respectively) intraperitoneally and LPS was instilled intratracheally (2 mg/kg of body weight), facilitated by a laryngoscope. Four hours after LPS instillation, mice were divided into groups and administered 50 µl of PBS or EVs isolated from 10^6^ of MSCs via tail vein. Mice were euthanised and BALF was taken for analysis 24 hours after LPS administration.

### Statistical analysis

Statistical analysis was performed using Prism V.7 software (GraphPad, USA). Experiments were done at least in triplicate, the average of three technical replicates was taken as a single data point for each MDM donor, and the points were pooled together for statistical analysis. Data were presented as the mean with SD. Mann-Whitney U test or Kruskal-Wallis test with Dunn’s selected comparisons were used. Statistical significance level was set at p<0.05.

## Results

### MSCs limit pro-inflammatory responses of human macrophages via the upregulation of phosphorylated STAT5-SOCS1 signalling

**Figure 1:**
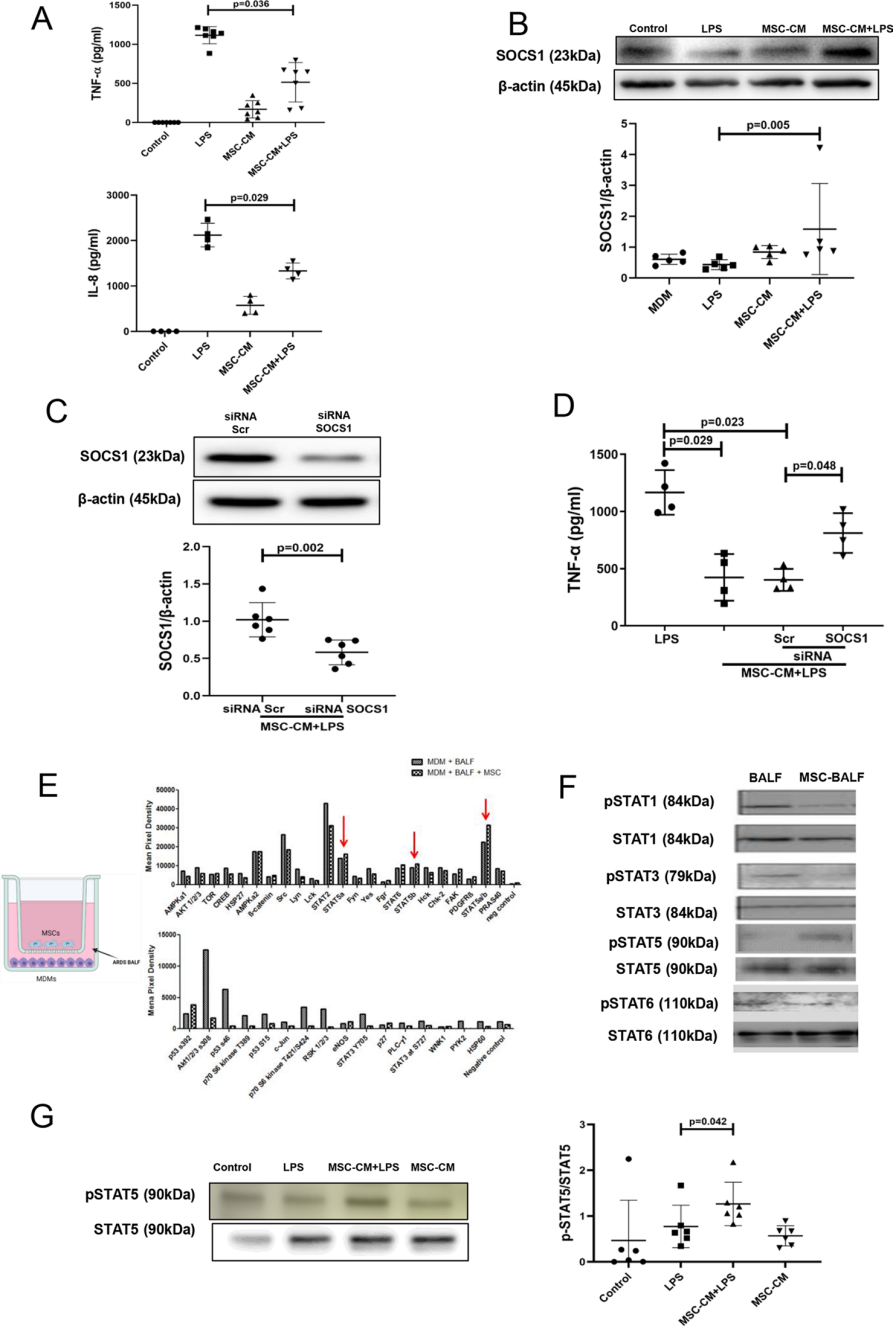
Upregulation of SOCS1 protein expression in human macrophages is critical for the paracrine effect of MSCs. SOCS1 upregulation is accompanied by the activation of STAT5 phosphorylation. (A) Levels of TNF-α and IL-8 in MDM conditioned medium after 24 hours of exposure to LPS (measured by ELISA) (n=4–7). (B) Immunoblot for protein expression levels of SOCS1 and β-actin in human MDM lysates after stimulation with LPS for 24 hours. Immunoblots were quantified by densitometry and normalised using β-actin expression (n=5). (C) Immunoblot of human MDM lysates, after MDMs were transfected with SOCS1 or scrambled siRNA and stimulated with LPS for 24 hours. Immunoblots were quantified by densitometry and normalised using β-actin expression (n=6). (D) Levels of TNF-α in the conditioned medium of MDMs transfected with scrambled or SOCS siRNA after LPS stimulation for 24 hours, measured by ELISA (n=4). (E) On the left, schematic image showing the co-culture of human MDMs with MSCs using a Transwell system which involved exposure to patient with ARDS bronchoalveolar lavage fluid (BALF) for 24 hours. On the right, phosphokinase array data from human MDMs cell lysates after exposure to ARDS BALF with and without MSC co-culture. Red arrows showed only STAT5a and STAT5b in MDMs could be upregulated by MSCs (n=1). (F) Immunoblots of different STAT proteins in human MDM cell lysates. (G) Immunoblot of pSTAT5 and STAT5 in human MDM lysates at 24 hours after MSC-CM treatment and LPS stimulation. Immunoblots of pSTAT5 were quantified by densitometry and normalised using total STAT5 protein expression (n=6). Data are represented as mean±SD. Kruskal-Wallis test with post-hoc Dunn’s test (A, B, D, G), Mann-Whitney test (C). ARDS, acute respiratory distress syndrome; CM, conditioned medium; IL, interleukin; LPS, lipopolysaccharide; MDM, monocyte-derived macrophages; MSC, mesenchymal stromal cells; pSTAT, phosphorylated STAT; siRNA, small interfering RNA; SOCS1, suppressor of cytokine signalling 1; STAT, signal transducers and activators of transcription; TNF, tumour necrosis factor.

Human MDMs were exposed to LPS with or without the presence of MSC CM for 24 hours. As expected, LPS stimulation resulted in the robust upregulation of tumour necrosis factor (TNF)-α and interleukin (IL)-8 secretion by MDMs, and these responses were significantly ameliorated by MSC CM (figure 1A). At the same time, MSC CM induced significant upregulation of SOCS1 protein expression levels in macrophages in the presence of LPS (figure 1B). To corroborate the role of SOCS1 in macrophage modulation by MSCs, SOCS1 expression in MDMs was silenced by transfection with small interfering (siRNA). siRNA transfection resulted in approximately 60% downregulation of SOCS1 protein expression (figure 1C). Transfection with SOCS1 siRNA but not with scrambled siRNA abrogated the ability of MSC CM to downregulate LPS-induced TNF-α secretion by MDMs, suggesting that SOCS1 is a critical mediator of MSC modulation of macrophages (figure 1D). SOCS proteins act as feedback inhibitors of JAK-STAT signalling and their expression is activated by interaction with phosphorylated STATs (pSTATs). In order to investigate which of the STATs was activated by MSC CM in MDMs in the setting of human ARDS, we co-cultured MDMs with MSCs on Transwell inserts, and exposed them to the samples of BALF from patients with ARDS. After 24 hours of BALF exposure, macrophages were lysed and the cell lysates subjected to a membrane-based phospho-kinase antibody array. Interestingly, it was observed that among four different STATs included in the array, only STAT5a/5b phosphorylation was increased in macrophages by MSCs in the presence of ARDS BALF. Notably, STAT2, STAT3 and STAT6 were not activated by MSCs (figure 1E). These results were further confirmed by Western blotting for STAT3, STAT5 and STAT6. We also probed for STAT1 phosphorylation, which was not included in the array, and found that pSTAT1 was also downregulated by MSCs (figure 1F). Furthermore, stimulation of MDMs with LPS in the presence of MSC CM led to significant pSTAT5 upregulation (figure 1G).

### EVs but not EV-free CM recapitulate effects of complete MSC CM on pSTAT5 and SOCS1 expression in human MDMs

**Figure 2:**
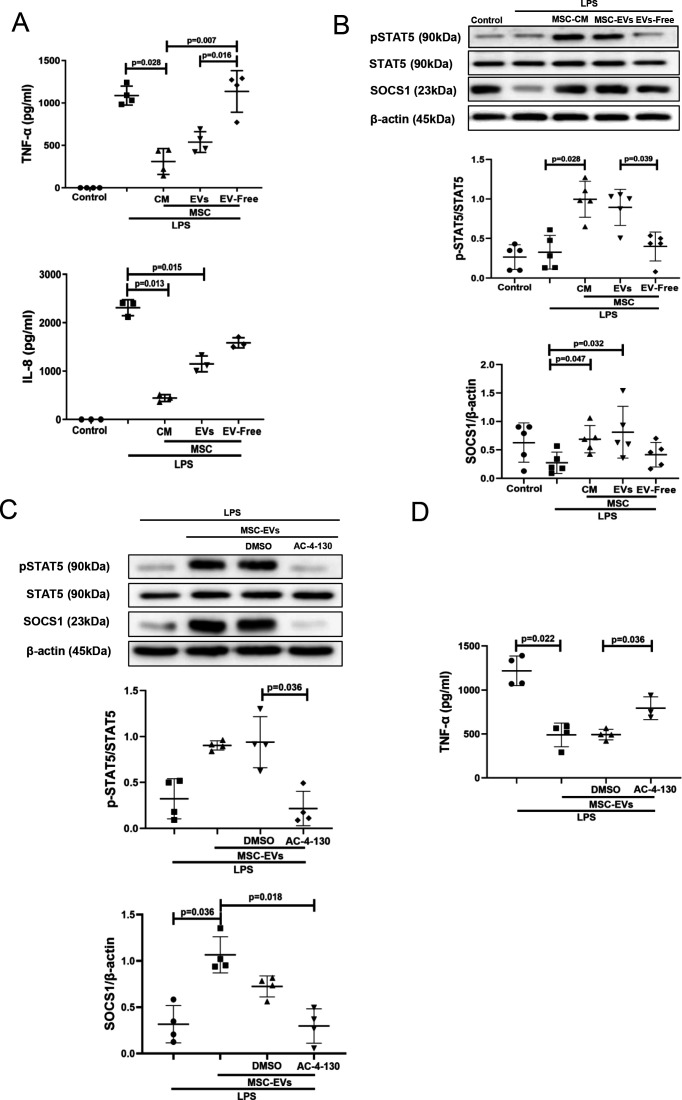
MSC-EVs but not EV-free MSC conditioned medium are responsible for the inhibition of LPS-induced cytokine secretion and upregulation of pSTAT5 and SOCS1 expression in human MDMs. STAT5 phosphorylation is critical for SOCS1 upregulation by EVs. (A) Levels of TNF-α and IL-8 in MDM conditioned medium (measured by ELISA) after LPS stimulation for 24 hours (n=3–4). (B) Immunoblot for protein expression levels of pSTAT5, STAT5, SOCS1 and β-actin in human MDMs lysates after stimulation with LPS for 24 hours. Immunoblots were quantified by densitometry and normalised using STAT5 protein expression for pSTAT5 or β-actin for SOCS1 (n=5). (C) Immunoblot of pSTAT5, STAT5, SOCS1 and β-actin in MDM lysates after MDMs were pre-treated with pharmacological STAT5 inhibitor AC-4–130. Immunoblots were quantified by densitometry and normalised using total STAT5 expression levels for pSTAT5 or β-actin expression levels for SOCS1 (n=4). (D) Levels of TNF-α in MDM conditioned medium after pre-treatment with STAT5 inhibitor and exposure to LPS for 24 hours (n=3–4). Data are represented as mean±SD. Kruskal-Wallis test with post-hoc Dunn’s test (A, B, C, D). CM, conditioned medium; DMSO, dimethyl sulfoxide; IL, interleukin; EVs, extracellular vesicles; IL, interleukin; LPS, lipopolysaccharide; MDM, monocyte-derived macrophages; MSC, mesenchymal stromal cells; pSTAT, phosphorylated STAT; SOCS1, suppressor of cytokine signalling 1; STAT, signal transducers and activators of transcription; TNF, tumour necrosis factor.

The next step was to identify which fraction of the MSC CM (EVs or soluble mediators) was responsible for the observed effects on macrophage modulation. EVs were isolated from MSC CM by ultracentrifugation as previously described^29^ and characterised according to guidelines of International Society for Extracellular Vesicles (ISEV) (online supplemental figure 1A-D). EV supernatants after ultracentrifugation were subjected to nanoparticle tracking analysis, which has confirmed their depletion from EVs (online supplemental figure 1E). Macrophages were stimulated with LPS and co-cultured with MSC CM, EVs or EV-free supernatants for 24 hours. Interestingly, EVs and MSC CM were comparably effective in downregulation of LPS-induced TNF-α and IL-8 secretion levels (figure 2A) and also in stimulation of pSTAT5 and SOCS1 activation in MDMs (figure 2B), while EV-free CM had no effect. Thus, we decided to further focus on MSC EVs as the active component of MSC CM. To further investigate the role of STAT5 signalling in MDMs, AC-4–130, a specific pharmacological STAT5 inhibitor was used.^33^ AC-4–130 treatment of MDMs abolished the effect of MSC EVs on SOCS1 upregulation (figure 2C) and pro-inflammatory cytokine secretion (figure 2D).

### MSC EVs modulate pro-inflammatory cytokine secretion and pSTAT5-SOCS1 signalling in MDMs in the presence of plasma from patients with ARDS

**Figure 3:**
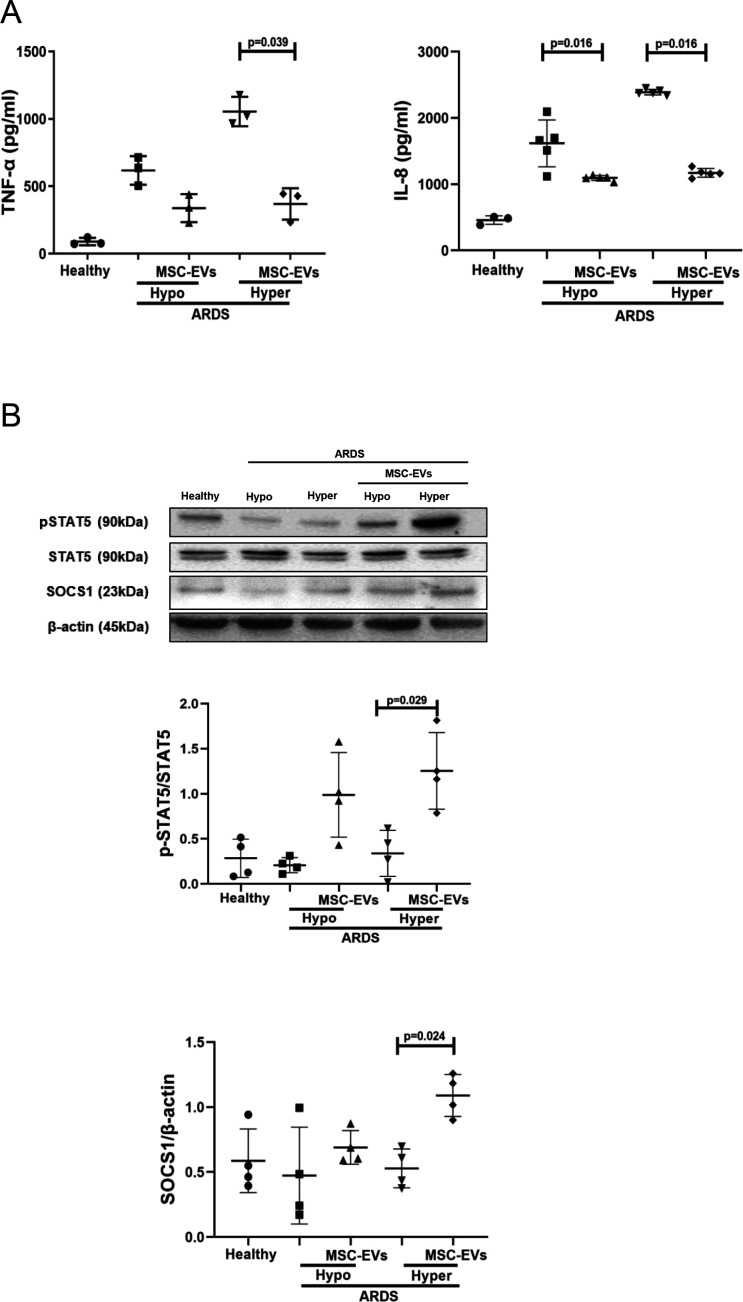
MSC EVs regulate pro-inflammatory cytokine secretion and pSTAT5 and SOCS1 expression in MDMs exposed to plasma samples from patients with ARDS in vitro. (A) Levels of TNF-α and IL-8 in MDMs supernatants (measured by ELISA) after stimulation with pooled healthy or hypo-inflammatory or hyper-inflammatory ARDS plasma for 24 hours (n=3–5). (B) Immunoblot of pSTAT5, STAT5, SOCS1 and β-actin protein expression in human MDM lysates after MDM exposure to healthy or ARDS plasma for 24 hours (n=4). Immunoblots were quantified by densitometry and normalised using total STAT5 expression for pSTAT5 or β-actin expression for SOCS1. Data are represented as mean±SD. Kruskal-Wallis test with post-hoc Dunn’s test (A, B). ARDS, acute respiratory distress syndrome; CM, conditioned medium; IL, interleukin; EV, extracellular vesicles; IL, interleukin; MDM, monocyte-derived macrophages; MSC, mesenchymal stromal cells; pSTAT, phosphorylated STAT; SOCS1, suppressor of cytokine signalling 1; STAT, 5, signal transducers and activators of transcription 5; TNF, tumour necrosis factor.

To test if this mechanism of macrophage modulation is relevant in the human ARDS environment, and to investigate EV effects in the different ARDS phenotypes, MDMs were cultured for 24 hours in the presence of plasma samples of patients with ARDS which were previously classified into hypo-inflammatory and hyper-inflammatory phenotypes.^7^ In this model, macrophage exposure to both types of ARDS plasma elicited robust upregulation of TNF-α and IL-8 secretion, and MSC EVs were capable of significant alleviation of pro-inflammatory cytokine production in the presence of both types of ARDS plasma (figure 3A). This effect was coupled with EV activation of the pSTAT5-SOCS1 signalling pathway in both ARDS environments, reaching statistical significance in the presence of hyper-inflammatory plasma (figure 3B).

### The effect of MSC EVs on MDM modulation is mediated by transfer of miR-181a which regulates phosphatase and tensin homolog-pSTAT5-SOCS1 axis

**Figure 4:**
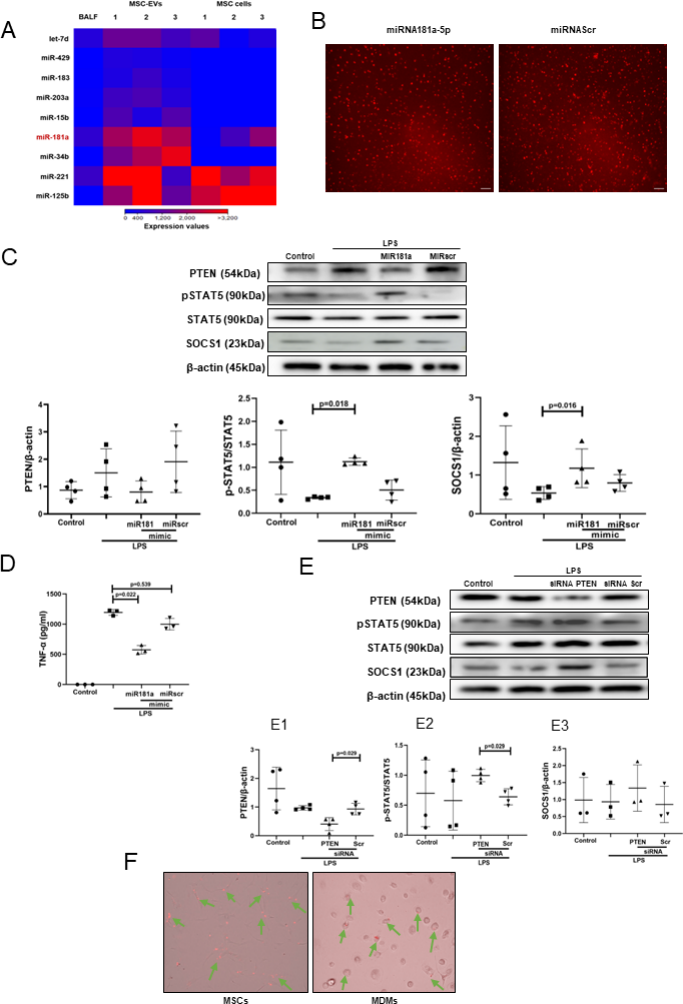
Transfer of miRNA-181a-5p in MSC EVs modulates LPS-induced secretion of pro-inflammatory cytokines through PTEN-pSTAT5-SOCS1 axis. (A) Heat map of next generation sequencing data comparing BALF, cell and MSC-EV expression of selected micro RNAs. ‘1’, ‘2’, ‘3’ labelling refers to different MSC donors. (B) Representative live microscopy images of human MDMs transfection with Dy574-labelled miRNA181a-5p mimic (left) and miRNA Scramble/Negative control mimic (right) as an indicator of efficiency of transfection, monitored by immunofluorescence. Images were taken using EVOS FL Auto epifluorescent microscope (Objective lens 10×, scale bar=50 µm). (C) Immunoblot of PTEN, pSTAT5, STAT5, SOCS1 and β-actin in human MDMs lysates transfected with miRNA181a-5p mimic and miRNA Scramble (negative control) mimic after LPS stimulation for 24 hours. Immunoblots were quantified by densitometry and normalised using total STAT5 expression for pSTAT5 or β-actin expression for PTEN/SOCS1 (n=4). (D) Levels of TNF-α secretion by MDMs after stimulation with LPS for 24 hours (n=3). (E) Immunoblot of PTEN, pSTAT5, STAT5, SOCS1 and β-actin in human MDMs lysates after MDMs were stimulated with LPS for 24 hours. Immunoblots were quantified by densitometry and normalised using total STAT5 expression for pSTAT5 or β-actin expression for PTEN/SOCS1 (n=3–4). (F) Representative live microscopy images of MSCs (left) and human MDMs (right) transfection with Dy574-labelled miRNA181a-5p mimic. The images were taken using EVOS FL Auto epifluorescent microscope (Objective lens 40×, scale bar=50 µm). Data are represented as mean±SD. Kruskal-Wallis test with post-hoc Dunn’s test (C, D), Mann-Whitney test (E). EV, extracellular vesicles; LPS, lipopolysaccharide; MDM, monocyte-derived macrophages; miRNA, micro RNA; MSC, mesenchymal stromal cells; pSTAT, phosphorylated STAT; PTEN, phosphatase and tensin homolog; siRNA, small interfering RNA; SOCS1, suppressor of cytokine signalling 1; STAT, 5, signal transducers and activators of transcription 5; TNF, tumour necrosis factor.

To explore the miRNA contents of MSC EVs produced by MSCs in the ARDS environment, MSCs were stimulated with ARDS BALF for 24 hours, EVs isolated and subjected to small RNA sequencing. Analysis revealed that EVs express up to 284 known miRNAs as identified in miRBase V.21. Of those, 20 miRNAs were found to be significantly enriched in EVs compared with their parent MSCs, suggesting that these are selectively incorporated into EVs during EV maturation in the ARDS environment. Notably, miR-181a was among the most highly expressed miRNAs in EVs compared with parent MSCs and BALF (figure 4A), its expression in EVs was further confirmed by RT-PCR (data not shown). We were particularly intrigued by miR-181a because one of its well-established target genes is phosphatase and tensin homolog (PTEN),^34^ which, among other important regulatory roles, negatively regulates STAT5 signalling.^35 36^

To investigate whether miR-181a alone can modulate MDMs, we transfected MDMs with Dy574-labelled miR181a-5p mimic (efficiency of transfection was monitored by immunofluorescence) (figure 4B). In the presence of LPS, overexpression of miR-181a in MDMs resulted in downregulation of PTEN and significant upregulation of pSTAT5 and SOCS1 protein expression levels (figure 4C) coupled with significantly decreased TNF-α production by macrophages (figure 4D).

To confirm the critical role of PTEN for the activation of pSTAT5-SOCS1 signalling pathway in MDMs, PTEN expression in MDMs was silenced using siRNA. Consistent with the above results, PTEN silencing resulted in the upregulation of pSTAT5 and SOCS1 protein levels in the presence of LPS (figure 4E). To further confirm that miR-181a could be transferred to MDMs via MSC EVs, MSCs were transfected with fluorescently labelled Dy574-miR181a-5p, EVs isolated and applied to MDM cultures. miRNA uptake was visualised by EVOS fluorescent microscopy (figure 4F).

### miR-181a-5p-PTEN-pSTAT5-SOCS1 pathway is responsible for MSC EV modulation of MDMs in the presence of ARDS plasma

**Figure 5:**
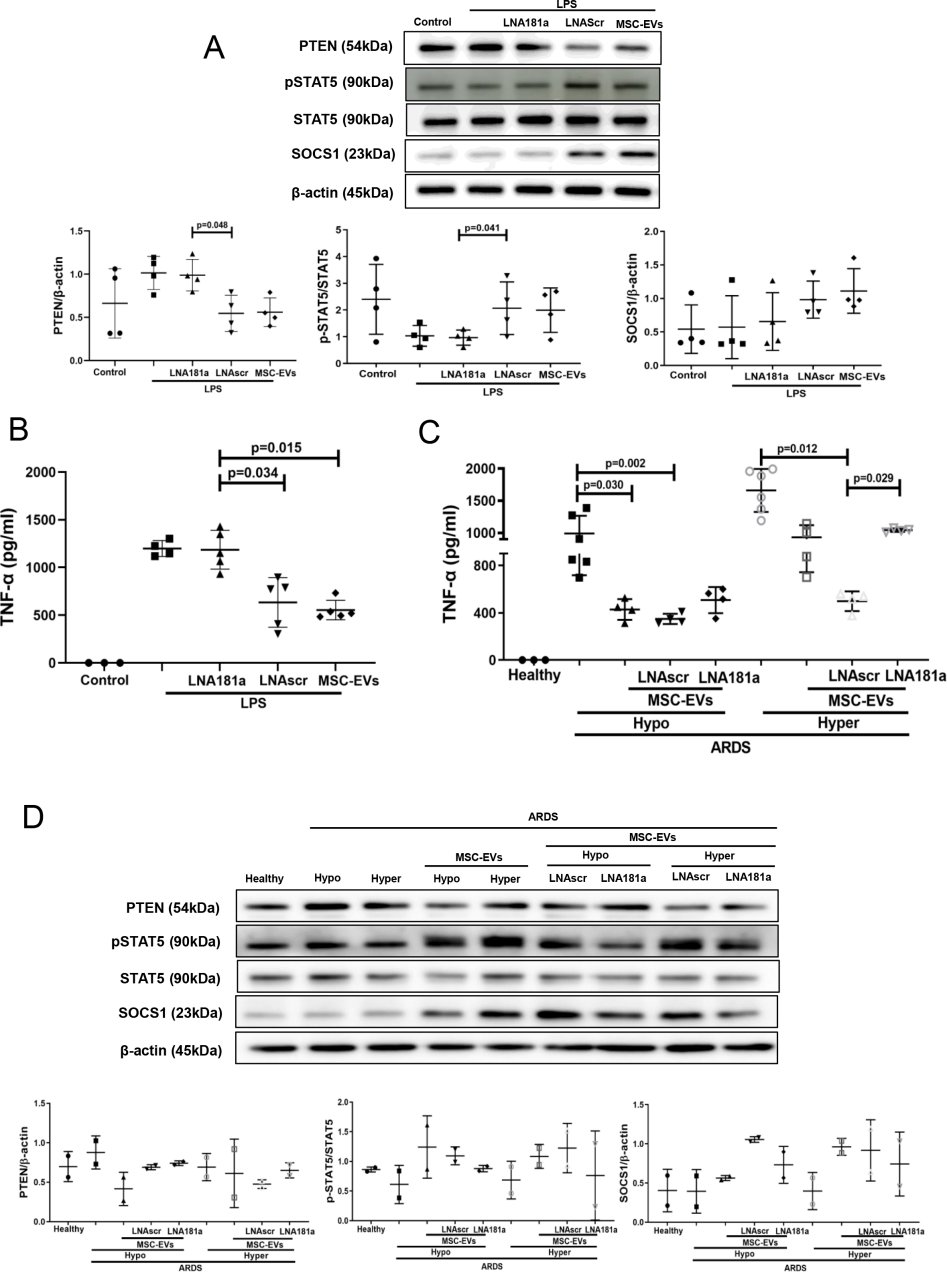
miRNA-181a transfer through MSC EVs negates the pro-inflammatory response in human MDMs when stimulated with LPS or hyper-inflammatory ARDS plasma. (A) Immunoblot of PTEN, pSTAT5, STAT5 and β-actin in human MDM lysates after stimulation with LPS for 24 hours. Immunoblots were quantified by densitometry and normalised using total STAT5 expression for pSTAT5 or β-actin expression for PTEN/SOCS1 (n=4). (B) Levels of TNF-α secretion by MDMs after stimulation with LPS for 24 hours (n=3–4). (C) Levels of TNF-α secretion by MDMs after stimulation with healthy or pooled hypo-inflammatory or hyper-inflammatory ARDS plasma for 24 hours (n=3–6). (D) Immunoblot of PTEN, pSTAT5, STAT5, SOCS1 and β-actin in human MDMs lysates after stimulation with healthy or hypo-inflammatory or hyper-inflammatory ARDS plasma for 24 hours. Immunoblots were quantified by densitometry and normalised using total STAT5 expression for pSTAT5 or β-actin expression for PTEN/SOCS1 (bottom panel, n=2). Data are represented as mean±SD. Kruskal-Wallis test with post-hoc Dunn’s test (A, B, C). ARDS, acute respiratory distress syndrome; EVs, extracellular vesicles; LNA, locked nucleic acid; LPS, lipopolysaccharide; MDM, monocyte-derived macrophages; miRNA, micro RNA; MSCs, mesenchymal stromal cells; pSTAT, phosphorylated STAT; PTEN, phosphatase and tensin homolog; SOCS1, suppressor of cytokine signalling 1; STAT, signal transducers and activators of transcription ;TNF, tumour necrosis factor.

Specific locked nucleic acid (LNA) 181a inhibitor was used to silence miR-181a-5p expression in MSCs.^16^ EVs isolated from MSCs transfected with LNA181a were unable to downregulate expression of PTEN, activate phosphorylation of STAT5 or upregulate SOCS1 expression, compared with EVs isolated from untransfected MSCs or MSC transfected with scrambled LNA inhibitor (figure 5A). Consistently, knockdown of miR-181a expression abrogated ability of the EVs to downregulate LPS-induced secretion of TNF-α by MDMs (figure 5B).

To further investigate if miR-181a-5p transfer in MSC EVs is relevant for MDM modulation in the human ARDS environment, MDMs were exposed to plasma from patients with ARDS for 24 hours (as in figure 3) and co-cultured with EVs, LNA181a EVs or scrambled LNA EVs. Interestingly, silencing of miR-181a in EVs (LNA181a EVs) significantly abrogated inhibitory effect of EVs on MDM TNF-α production only in the presence of hyper-inflammatory plasma (figure 5C). Activation of the PTEN-pSTAT-SOCS1 signalling pathway by EVs was comparably negatively affected in the LNA181a group in both types of ARDS plasma (figure 5D)

### Transfer of miR181a-5p in MSC EVs is critical for the EV immunomodulatory effect in vivo

**Figure 6:**
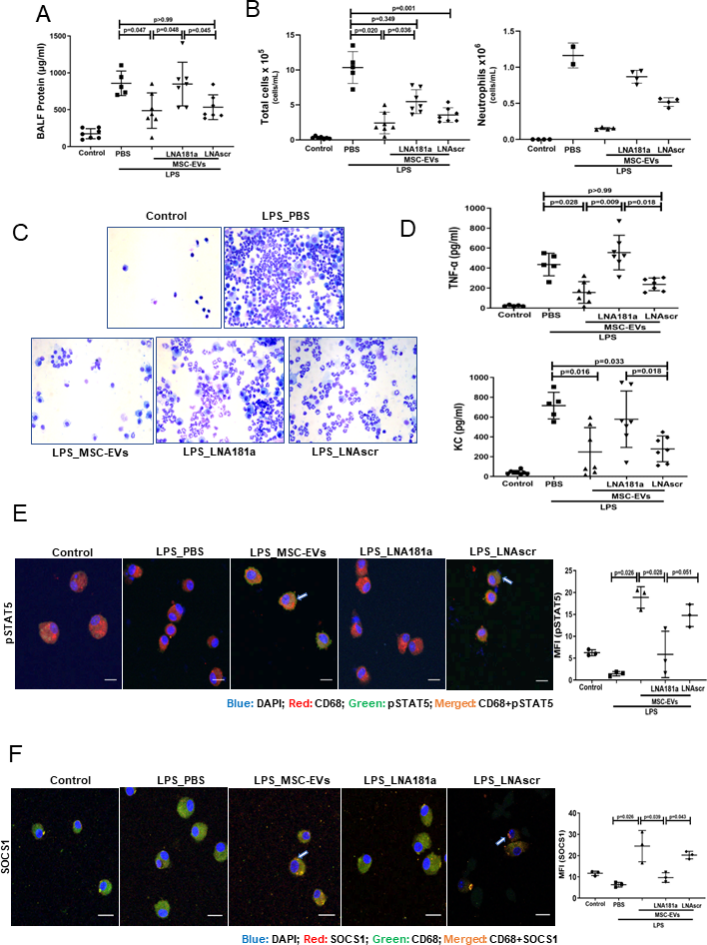
Transfer of miR181a is critical for the immunomodulatory effects of MSC-EVs in vivo. (A) Total protein concentrations in the BALF samples 24 hours after LPS administration (n=5–7 mice per group). (B) Total leucocyte counts (left graph) and neutrophil cell counts (right graph) in the BALF samples (total cells (n=5–7 mice per group); neutrophils (n=2–4 mice per group)). (C) Representative images of BALF cytospin preparations demonstrating cell recruitment to the airspaces 24 hours after LPS administration. Images were taken using Leica Epifluorescence DM5500 microscope (Objective lens ×20). (D) BALF levels of TNF-α and keratinocyte-derived chemokine (KC, murine analogue of interleukin-8) (n=5–7 mice per group). (E) Representative confocal microscopy of alveolar macrophages, isolated from BALF 24 hours after LPS administration and stained with anti-CD68 (alveolar macrophage marker) and anti-pSTAT5 Ab. Arrows indicate co-localisation of CD68 and pSTAT5. The images were taken using Leica SP8 confocal microscope with a 100× oil-immersion objective (n=3, scale bar=50 µm). Quantitative fluorescence intensity was analysed by Image J software (MFI-pSTAT5). (F) Representative confocal microscopy of alveolar macrophages, isolated from BALF samples 24 hours after LPS administration and stained with anti-CD68 and anti-SOCS1 Ab. Arrows indicate co-localisation of CD68 and SOCS1. Images were taken using Leica SP8 confocal microscope with a 100× oil-immersion objective (scale bar=50 µm). Quantitative fluorescence intensity was analyses by Image J software (MFI-SOCS1). Data are represented as mean±SD. Kruskal-Wallis test with post hoc Dunn’s test (A, B, D, E, F). Ab, antibody; BALF, bronchoalveolar lavage fluid; DAPI, 4',6-diamidino-2-phenylindole; EVs, extracellular vesicles; LNA, locked nucleic acid; LPS, lipopolysaccharide; MFI, mean fluorescent intensity; MSCs, mesenchymal stromal cells; PBS, phosphate-buffered saline; pSTAT, phosphorylated STAT; SOCS1, suppressor of cytokine signalling 1; STAT, signal transducers and activators of transcription ;TNF, tumour necrosis factor.

To induce lung injury, C57BL/6 mice were instilled with LPS intratracheally and 4 hours later treated with PBS, EVs, LNA181a EVs or scramble LNA EVs via tail vein. LPS instillation resulted in significant lung injury and inflammatory cell infiltration into alveolar spaces as demonstrated by increased protein levels in the BALF (figure 6A), BALF total inflammatory cell and absolute neutrophil counts (figure 6B,C) as well as increased levels of inflammatory cytokines TNF-α and keratinocyte (KC) (figure 6D). While control EVs and LNAscr EVs were able to comparably reverse these effects, LNA181a EVs were not effective.

To investigate if EVs were able to activate pSTAT5-SOCS1 signalling in vivo, in the same experiments, murine alveolar macrophages (AMs) were isolated from BALF at 20 hours after EV treatment, seeded onto the chamber slides (Thermo Fisher), fixed and expression levels of pSTAT5 and SOCS1 in AMs was investigated by immunofluorescence. Consistently with the in vitro results, LPS injury resulted in downregulation of pSTAT5 and SOCS1 expression levels in AMs, which was restored by control EVs and LNAscr EVs, however the restoration was not evident in animals which received LNA181a EVs (figure 6E and F).

### Overexpression of miRNA181a-5p in MSCs enhances the therapeutic efficacy of MSC EVs in vivo

**Figure 7:**
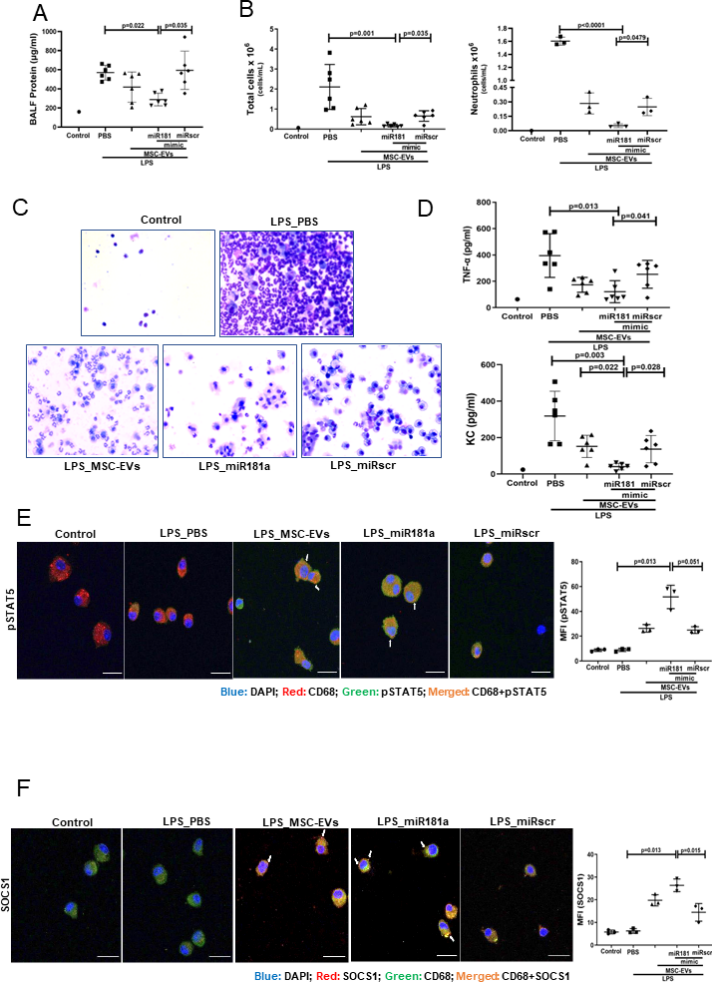
Overexpression of miR181a in MSC-EVs improves the therapeutic efficacy of MSC-EVs in the in vivo model of LPS-induced lung injury. (A) Total protein concentrations in the BALF samples 24 hours after LPS administration (n=6 mice per experimental group, n=1 sham control). (B) Total leucocyte counts (left graph, n=6 mice per experimental group, n=1 sham control) and neutrophil cell counts (n=1–3, right graph) in the BALF samples). (C) Representative images of the BALF cytospin preparations, 24 hours after LPS administration demonstrating cell recruitment to the airspaces. Images were taken using Leica Epifluorescence DM5500 microscope (objective lens original magnification ×20). (D) BALF levels of TNF-α and keratinocyte-derived chemokine (KC, murine analogue of interleukin-8) (n=6 mice per experimental group, n=1 sham control). (E) Representative confocal microscopy of alveolar macrophages, isolated from BALF 24 hours after LPS administration and stained with anti-CD68 and anti- pSTAT5 Ab. Arrows indicate co-localisation of CD68 and pSTAT5. The images were taken using Leica SP8 confocal microscope with a 100× oil-immersion objective (n=3, scale bar=50 µm). Quantitative fluorescence intensity was analysed by Image J software (MFI-pSTAT5). (F) Representative confocal microscopy of alveolar macrophages stained with anti-CD68 and anti-SOCS1 Ab. Arrows indicate co-localisation of CD68 and SOCS1. The images were taken using Leica SP8 confocal microscope with a 100× oil-immersion objective (scale bar=50 µm). Quantitative fluorescence intensity was analysed by Image J software (MFI-SOCS1). Data are represented as mean±SD. Kruskal-Wallis test with post-hoc Dunn’s test (A, B, D, E, F). Ab, antibody; BALF, bronchoalveolar lavage fluid; DAPI, 4',6-diamidino-2-phenylindol; EVs, extracellular vesicles; LPS, lipopolysaccharide; MFI, mean fluorescent intensity; MSCs, mesenchymal stromal cells; PBS, phosphate-buffered saline; pSTAT, phosphorylated STAT; SOCS1, suppressor of cytokine signalling 1; STAT, signal transducers and activators of transcription ;TNF, tumour necrosis factor.

MSCs were transfected with miR-181a mimic (or scrambled mimic), EVs isolated and administered as a treatment to LPS-injured mice as above. EVs isolated from miR-181a overexpressing MSCs demonstrated significantly stronger ability to reduce lung injury compared with miRscr EVs as indicated by protein levels in the BALF (figure 7A). In addition, miRNA181a-overexpressing EVs had a significantly stronger effect on the reduction of inflammatory cell infiltration (figure 7B and C) and BALF levels of TNF-α and KC cytokines (figure 7D) compared with miRscr EVs.

In line with these results, administration of the miR-181a-overexpressing EVs was accompanied by significantly more pronounced activation of pSTAT5-SOCS1 expression in AM compared with control EVs and scrambled EVs administration groups (figure 7E and F).

## Discussion

The main findings of this study can be summarised as follows:

1. SOCS1 protein controls the anti-inflammatory state of human macrophages in the ARDS environment. The MSC effect on macrophage modulation is mediated through upregulation of SOCS1 expression in macrophages.
2. MSC EVs are principally responsible for the MSC paracrine effects on human macrophage modulation.
3. The miRNA181a-PTEN-pSTAT5-SOCS1 axis is crucial for MSC EV anti-inflammatory modulation of human MDMs exposed to LPS and murine AMs in vivo as shown in the mouse model of LPS-induced lung injury.
4. While MSC EVs robustly alleviate MDM excessive secretion of pro-inflammatory cytokines in the presence of ARDS plasma regardless of ARDS phenotype, miR-181-a transfer is crucial for the anti-inflammatory effect of MSC EVs only in the hyper-inflammatory plasma
5. The overexpression of miRNA181a in MSCs enhances therapeutic efficacy of MSC EVs in vivo.

Macrophages play a central role in the orchestration of the innate immune response. Dysregulated macrophage function drives pathogenesis of many diseases, including ARDS.^12 13^ Identification of the molecular mechanisms that counteract inappropriate macrophage activation would improve our understanding of the disease and may reveal new therapeutic targets. SOCS proteins have attracted a lot of interest as potent regulators of the innate immune response and potential targets for therapeutic manipulation. SOCS1 is an essential negative regulator of pro-inflammatory signalling in antigen-presenting cells that negatively regulate toll-like receptor signalling.^37–39^ Its relevance as critical immune checkpoint molecule has been recently highlighted by Wang *et al*, a study that revealed that silencing of SOCS1 in dendritic cells (DC) improves the efficacy of a DC based anti-leukaemia vaccine in a Phase I–II clinical trial.^40^ In macrophages, in addition to aforementioned mechanisms, SOCS1 protein was also shown to contribute to metabolic reprogramming through inhibition of rate limiting glycolytic enzymes.^25^ However, to date SOCS proteins have been predominantly studied in animal models and understanding of the SOCS1 role in regulation of human macrophages remains limited. Here, we for the first time, report that SOCS1 expression in primary human macrophages is downregulated after inflammatory stimulation with LPS or plasma from patients with ARDS and restored by MSC CM or MSC EVs. Notably, SOCS1 upregulation is critical to the MSC or MSC EV ability to reprogramme macrophages towards less inflammatory state, as the anti-inflammatory effect of MSCs or EVs is lost when SOCS1 expression in macrophages is silenced. These findings are in line with Prele *et al* 41 who demonstrated that SOCS1 was critical for inhibition of sustained LPS-induced TNF-α secretion by human monocytes. In addition, SOCS1 messenger RNA (mRNA) expression was found to be downregulated in AMs and lung tissues of patients with COPD, where mRNA levels were found to be negatively correlated with BALF TNF-α levels and positively correlated with forced expiratory volume in 1 s.^42^ Another study revealed that SOCS1 expression was reduced in the bronchial epithelium of patients suffering from severe asthma.^43^ Interestingly, SOCS1 activation has been identified by in silico modelling approach as a strategy for intervention in sepsis.^44^

SOCS proteins are generally induced through the JAK/STAT pathway following cytokine stimulation,^45^ phospho-STAT then dimerises and translocates to the nucleus where it upregulates the expression of target genes. Phospho-STAT likely acts directly on the gene promoters for CIS and SOCS1-3 proteins.^46^ Ghazawi *et al* have established that in primary CD8 T lymphocytes, induction of SOCS1 is dependent on the pSTAT5 signalling,^47^ however, if a similar mechanism exists in human macrophages and whether it contributes to the MSC effect, was not known. We observed that pSTAT5 is the only STAT which was activated in macrophages by MSCs, when co-cultures of MDMs and MSCs were exposed to ARDS BALF (as a surrogate of ARDS environment) (figure 1E,F). Similarly, pSTAT5 was downregulated by LPS and restored by MSCs (figure 1G). More critically, pharmacological inhibition of STAT5 abrogated effect of MSCs or MSC EVs on SOCS1 upregulation, confirming that MSCs upregulate SOCS1 through activation of the pSTAT5 pathway (figure 2C,D). It is important to note here that the *socs1* promoter contains STAT1-, STAT3- and STAT6-binding sites.^48 49^ Several previous studies, both in vitro and in transgenic animals have shown links between SOCS1 induction and pSTAT1, pSTAT3 or pSTAT6 signalling.^23^ However, in our in vitro models, we were not able to detect changes in STAT6 activation in MDMs between any of the experimental groups, whereas STAT1 and STAT3 phosphorylation was inversely correlated with SOCS1 expression (figure 1E,F).

EVs have emerged as a key factor responsible for the therapeutic effects of MSCs in lung injury, accumulating evidence, including our own studies, demonstrates that EVs are able to recapitulate many of the effects of the cell therapy.^17 29 50 51^ In agreement with these findings, in the present study, EVs but not EV-free MSC CM recapitulated the effect of the MSC co-culture on macrophage secretion of TNF-α and IL-8 and activation of pSTAT5-SOCS1 axis in the presence of LPS and, most importantly, when exposed to the plasma from patients with ARDS (figures 2 and 3).

Further, we sought to decipher what EV cargo is responsible for the observed effects.

We considered that the mechanism might be mediated by the transfer of miRNA. It has been previously demonstrated that MSCs behave differently in healthy and inflamed lung microenvironments.^52^ To find out which miRNAs are enriched in MSC EVs in the ARDS environment, we stimulated MSCs with BALF samples from patients with ARDS. MiRNA targeted sequencing demonstrated that number of miRNAs were significantly enriched in MSC EVs compared with their parent MSCs and BALF itself. miR-181a was among miRNAs significantly upregulated in the EVs (figure 4A). It has been previously demonstrated that this miRNA inhibits expression of PTEN in natural killer and T cells, while PTEN itself is a negative inhibitor of STAT5 phosphorylation.^34 35^ Further investigation confirmed that transfection of human MDMs with miR-181a mimic resulted in downregulation of PTEN expression and recapitulated effect of MSC and MSC EVs on macrophage modulation, whereas silencing of PTEN in macrophages or silencing of miR-181a expression in MSCs abrogated the observed effect (figure 4C–G). Collectively, these data suggest that MSC EVs modulate macrophages through the miR-181a-PTEN-pSTAT5-SOCS1 axis. It is important to highlight that this is a novel mechanism of SOCS1 activation which bypasses JAK mediated signalling. Furthermore, we have demonstrated that this mechanism is also relevant for macrophage modulation in the context of human ARDS microenvironment. Interestingly, although MSC EVs demonstrated potent anti-inflammatory effect in both phenotypes, EV mediated activation of pSTAT5-SOCS1 signalling and miR-181a transfer had stronger effects on macrophages in the presence of hyper-inflammatory plasma (figures 3 and 5). These findings warrant further investigation into the mechanisms responsible for such differential MDM responses. One may speculate that because hyper-inflammatory and hypo-inflammatory plasma samples were phenotyped based on the levels of soluble tumour necrosis factor recptor-1 (sTNFr-1), IL-6 and bicarbonate,^7^ the differences in the MDM responses to EVs could be attributed to different signalling induced by combination of these factors.

To confirm the importance of this mechanism for lung injury in vivo, endotoxin-injured mice were given MSC EVs isolated from control MSCs or from MSCs where miR-181a expression was inhibited by specific LNA inhibitor. While control EVs demonstrated significant therapeutic effect in this model, consistently with our previous report,^29^ EVs lacking miR-181a were not able to reduce lung injury and inflammatory cell infiltration into the lungs (figure 6A–D). Notably, administration of control EVs was coupled with significant upregulation of pSTAT5 and SOCS1 expression in AMs, while this effect was not present in the LNA181a EV treated group (figure 6E,F). These data corroborate our previous findings demonstrating that AMs are cellular mediators of the MSC and MSC EV effects.^16 17^ Moreover, these data contribute to the growing body of evidence that suggests the potential of MSC-derived EVs as a cell-free therapy for ARDS.^19 29 53^

To investigate if overexpression of miR-181a could enhance therapeutic efficacy of MSC EVs in vivo, mice were given EVs isolated from MSCs transfected with miRNA mimic or control EVs, isolated from MSCs transfected with scrambled miRNA. miR-181a overexpression resulted in significant augmentation of the therapeutic efficacy of EVs in this model (as indicated by reduction in BALF protein concentration and indices of pulmonary inflammation) (figure 7). Interestingly, Wei and colleagues have reported that overexpression of miR-181a in MSC exosomes enhanced their efficacy in the model of myocardial ischaemia-reperfusion injury. The mechanism of action was partially mediated through Treg polarisation by targeting the *c-Fos* gene.^54^ The anti-inflammatory role of miR-181 family in respiratory diseases has also been documented in several previous studies. Investigation of the whole-genome miRNA expression of bronchial airway epithelium from current smokers have found that miR-181d was decreased compared with never smokers.^55^

Our study has several limitations. We solely looked at pro-inflammatory cytokine production and did not explore other MSC EV effects on macrophages such as alterations in phagocytosis and metabolism, we also did not study functional contributions of other miRs (such as miR-34b, miR-125b, miR-203a) found to be upregulated in the EVs, these are being followed-up in ongoing studies. Stimulation of human macrophages for assessment of their phosphokinase activation profile and stimulation of MSCs for miRNA sequencing were performed using ARDS BALF, while investigation of the miR181a-PTEN-pSTAT5-SOCS1 pathway was conducted using ARDS plasma. Due to larger volumes of samples required for cell stimulation and subsequent EV isolation for sequencing it was not possible to use plasma in these experiments. The data obtained from BALF stimulation was used to provide an insight into the pathways activated in primary cells by ARDS alveolar microenvironment, which were then validated in plasma samples and in vivo. Although the use of the pooled samples for stimulations might reduce variability of responses, it is important to emphasise that we used MDMs from different donors and heterogeneity of plasma samples was reduced as they were phenotyped based on IL-6, sTNFr-1 and bicarbonate concentrations. In addition, the LPS-induced lung injury model is relatively mild and does not reflect all aspects of human ARDS. However, the primary goal of the in vivo experiments was to provide a proof of principle that the mechanism of MSC macrophage modulation via miR-181a transfer in EVs first identified in vitro in human cells is also relevant in vivo. Finally, statistical analyses of the majority (but not all) of in vitro experiments in this study are based on comparison of <5 observations. Despite the small sample size of observations in the individual experiments, we performed multiple independent gain-of-function and loss-of-function experiments in vitro and in vivo using different techniques which corroborated each other and substantiated the mechanism revealed in this study.

## Conclusions

**Figure 8:**
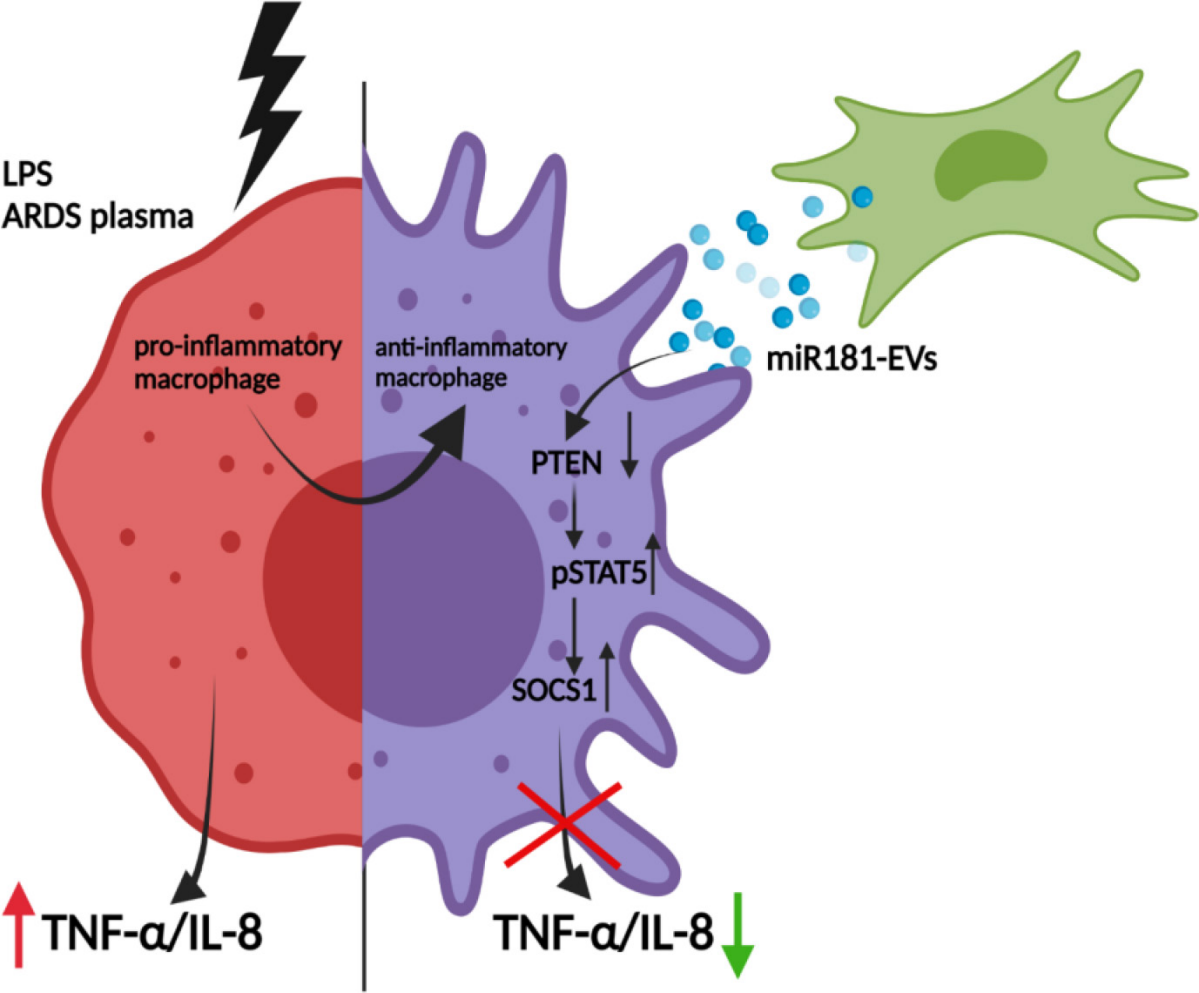
MSC EVs transfer miR-181a which modulates macrophage intracellular signalling through PTEN-pSTAT5-SOCS1 axis. Left: Stimulation of macrophage with inflammatory stimuli (LPS or ARDS plasma) results in the upregulation of secretion levels of pro-inflammatory cytokines TNF-α and IL-8. Right: miR181a packaged in MSC-EVs reprogrammes macrophage towards anti-inflammatory state through downregulation of PTEN and enhancement of pSTAT5/SOCS1 expression resulting in reduced levels of TNF-α and IL-8 production. ARDS, acute respiratory distress syndrome; EVs, extracellular vesicles; IL, interleukin; MSCs, mesenchymal stromal cells; PTEN, phosphatase and tensin homolog; pSTAT, phosphorylated STAT; SOCS1, suppressor of cytokine signalling 1; STAT, signal transducers and activators of transcription; TNF, tumour necrosis factor.

miRNA-181a transfer in MSC EVs is a novel mechanism of MSC anti-inflammatory modulation of macrophages through the PTEN-pSTAT5-SOCS1 axis, this pathway can be considered as a novel therapeutic target (figure 8). MSC EVs are capable of efficient macrophage reprogramming in the presence of inflammatory stimuli including hypo-inflammatory and hyper-inflammatory ARDS plasma. Overexpression of miRNA181a in MSCs may enhance their therapeutic efficacy in ARDS.

## Data Availability

Data are available upon reasonable request.
