# Supplementary material for: Mesenchymal stromal cells-derived extracellular vesicles reprogramme macrophages in ARDS models through the miR-181a-5p-PTEN-pSTAT5-SOCS1 axis
Source: Thorax. 2022 Aug 10;78(6):617–30. doi: 10.1136/thoraxjnl-2021-218194 (PMC10314068; doi:10.1136/thoraxjnl-2021-218194)
Supplement: Supplementary data [file thoraxjnl-2021-218194supp001.pdf]

## Methods and Materials:

### Cell Culture

Human Bone Marrow-derived Mesenchymal Stromal Cells (MSCs) were acquired from the Texas A&M University, Health Science Center, College of Medicine, Institute for Regenerative Medicine (Temple, TX) and the American Type Culture Collection (ATCC) (LGC Standards UK). These cells fulfil all requirements set by the International Society of Cellular Therapy (ISCT) for defining MSCs(1). MSCs were grown in MEM alpha with 16.5% foetal bovine serum, 100 U/ml penicillin, 100 µg/ml streptomycin and 2 mM L-Glutamine (Gibco). MSCs were used for experimentation at passages from 3 to 6. Primary human macrophages were isolated from buffy coat samples, seeded at 300,000 cells per well in 24-well plates and differentiated from monocytes for 5-7 days in the presence of 10 ng/ml granulocyte-macrophage colony-stimulating factor (GM-CSF) (R&D systems) in RPMI 1640 medium containing 10% foetal bovine serum, 100 U/ml penicillin and 100 µg/ml streptomycin. Buffy coats were obtained from the Northern Ireland Blood Transfusion Service (NIBTS). Ethical approval was granted by the School Research Ethics Committee of Queen's University Belfast. Multiple MSC and buffy coat donors were used in this study.

### MSC-CM Generation and Extraction of MSC-derived EVs

MSC conditioned medium was generated from MSCs grown to ~80% confluence (~600,000 cells) in T175cm$^2$ flasks or with 120,000 cells/well in 6-well plates. Cells were washed and media changed to RPMI 1640 with 1% foetal bovine serum for 24 hours before CM collection with centrifugation at 1500 rpm for 5 minutes at 4°C.

For MSC-EV isolation, MSCs were grown in T175cm$^2$ flasks or seeded at 200,000 cells/well in 6-well plates overnight. Cells were washed and media changed to serum free MEM alpha medium for 48 hours before collection. MSC-EVs were extracted from the serum free MSC-CM via centrifugation for 30min at 2000 x *g* at 4 °C, followed by ultracentrifugation (Type 70.1 Ti Fixed-Angle Titanium Rotor, Beckman Coulter) at 100,000 x *g* for 3 hours at 4 °C. MSC-EVs were resuspended in PBS (Gibco) in a volume relative to the number of cells used to generate the serum free MSC-CM (i.e., 25 µl PBS per 600,000 cells). MSC EVs were characterized according to the International Society for Extracellular Vesicles (ISEV)(2) guidelines as in or previous publication(3).

**Characterization of the morphology, size and surface marker expression of MSC-EVs**

MSC EVs were characterized according to the International Society for Extracellular Vesicles (ISEV) guidelines(2). Morphology was assessed using electron microscopy. MSC-EVs were isolated from $10^6$ cells and the EV pellet resuspended in 500 µl of 4% paraformaldehyde (PFA, ThermoFisher) and fixed for 30 minutes at RT. After fixation and washing the pellet three times, the EVs were resuspended in 30 µl PBS before 10 µl of each sample was added into formvar/carbon-coated grids (Science Services, München) for 20 minutes at RT. The grids were dried on filter paper (Whatman, UK) and fixed with 2% glutaraldehyde for 10 minutes. The grids were then washed in distilled water for 1 minute and subsequently dried. Next, a drop of 1% tannic acid ACS reagent (Sigma Aldrich, UK) was added for 40 minutes to stain mitochondrial membranes. Grids were then washed twice using PBS for 1 minute each, dried and stained with TAAB EM heavy metal stain 336 (TAAB, Laboratory Equipment LTD) for 30 minutes. Grids were then submerged in 50% ethanol (Sigma Aldrich) before being washed in distilled water. The morphology of MSC-EVs were visualized using transmission electron microscopy (TEM) (JEOL, JEM 1400Plus, Japan).

MSC-EV size distribution and concentration was assessed on a Nanoparticle tracking analysis (NTA) device, NanoSight NS300 (Malvern, UK). MSC-EVs were extracted from $10^6$ cells and diluted in distilled water to a final volume of 1 mL. Particles present in EVs were measured according to the NanoSight NS300 user manual with the detection threshold set to measure as many particles as possible. For each test, five 1-minute videos were recorded at 25°C and the NanoSight Software NTA was used for analysis.

MSC cell surface markers were assessed using flow cytometry and Western blotting. For Flow cytometry, MSC-EVs were extracted and coupled to 4 µm diameter aldehyde-sulfate latex beads (ThermoFisher Scientific) (100 µl of MSC-EVs were incubated with 0.25 µl of aldehyde/sulfate-latex beads (ø = 4 µm; $5.5 \times 10^6$ particles/ml) for 20 minutes at RT). Subsequently, 1 mL of 0.1% BSA (Sigma Aldrich) supplemented with 0.01% $NaN_3$ (G-Biosciences) was added to the sample and left overnight. Bead-coupled MSC-EVs were centrifuged at 2000 x *g* for 10 minutes, washed and centrifuged. The pellet was then stained with FITC Mouse anti human CD44 (BD Biosciences, #555478) and PE-Cy7 Mouse anti-human CD63 (BD Biosciences, #561982) for 45 minutes at 4°C. After the incubation, MSC-EVs were washed twice and resuspended in PBS for flow cytometry. Bead-coupled EVs and the respective isotype control antibody (negative control) were included in the flow cytometry experiments. Gating of EV-coupled beads were performed based on FCS/SSC parameters, with unbound EVs or possible antibody aggregates excluded from the analysis. Flow

cytometry was performed using a BD FACSCanto™ II flow cytometer using the FACSDiva software and data analysis was performed using FlowJo software (FlowJo, Ashland, OR).

For Western blotting, MSC-EVs were isolated from MSCs and protein extracted using 50µl 1X radioimmunoprecipitation assay (RIPA) (0.5M Tris-HCl, pH 7.4, 1.5M NaCl, 2.5% deoxycholic acid, 10% NP-40, 10mM EDTA, Millipore) containing protease and phosphatase inhibitors (Halt™ Protease and Phosphatase Inhibitor Cocktail, Thermo). The MSC-EVs were incubated at 4 ºC for 30 minutes and placed in an ice-cold sonication for 30 seconds. 25 µg of EV total protein was probed with primary antibody CD63 #MA5-32085 (1:1000, ThermoFisher) by Western blotting, further details below.

**Treatment of MDMs with MSC-CM/MSC-EVs**

Monocyte-derived macrophages (MDM) were treated with MSC-CM or MSC-EVs in the presence of *E. Coli* LPS O111:B4 (Millipore) at 10 ng/ml or 10% ARDS patients plasma from either a hypoinflammatory or hyperinflammatory subphenotype(4). Ethical approval for use of patient samples for laboratory research was obtained from the Office for Research Ethics Committees Northern Ireland.

**Western Blotting and ELISA**

Protein expression of PTEN, STAT5/phosphorylated STAT5 (pSTAT5) and SOCS1 was assessed by Western Blot. The total protein content of MDMs was quantified using the Pierce BCA Protein Assay Kit (Thermo Scientific). MDMs in 24 well plates were lysed using 50 µl RIPA containing protease and phosphatase inhibitors. Western blotting was carried out on the Mini-PROTEAN Tetra System (Bio-Rad). Separated proteins were transferred to PVDF membranes (Immun-Blot PVDF Membrane, Bio-Rad) before being blocked in 5% BSA in TBS and probed with primary antibodies at 1:1000 dilution in 5% BSA (in TBS-0.05% tween 20) overnight at 4ºC. Primary antibodies included: PTEN (D4.3) XP® Rabbit mAb #9188, Stat5 (D2O6Y) Rabbit mAb #94205, Phospho-Stat5 (Tyr694) Antibody #9351, SOCS1 (A156) Antibody #3950 and β-Actin (13E5) Rabbit mAb #4970 (Cell Signalling Technology). Membranes were subsequently probed with secondary antibody, anti-rabbit IgG HRP-linked Antibody #7074 at 1:2500 dilution in TBS-T (Cell Signalling Technology). Bands were visualised using the Clarity Western ECL Substrate (Bio-Rad) or SuperSignal West Femto Maximum Sensitivity Substrate (ThemoFisher Scientific) on the Chemi-XX6 G-Box (Syngene) using the GeneSys software. Densitometric analysis of bands were carried out using ImageJ 1.47v software (NIH). The relative density of the target protein and a loading control (β-actin

or unphosphorylated protein) were analysed and the signal ratios used to compare the difference of protein expression.

ELISAs were carried out on Nunc-Immuno 96 well MaxiSorp™ flat bottom plates (Merck). Proinflammatory cytokine secretion of tumor necrosis factor-α (Human TNF-α Duoset – DY210) and interleukin-8 (Human IL-8 Duoset - DY208) in cell supernatants were analysed as per the manufacturer's instructions (R&D). ELISAs were developed using TMB-I solution (Biopanda Diagnostics), with absorbances measured at 450 nm (with subtraction of correction reference wavelength of 570 nm) and data analysed by 4-paramater logistical regression.

**Membrane-based phospho-kinase antibody array**

The Proteome Profiler Array human phosphor-kinase array (R&D, ARY003B) kit was used to detect the phosphorylation of protein kinases. MDMs were seeded at 300,000 cells per well in 24 well plates and co-cultured with MSCs (60,000 per insert) seeded on 0.4μm transwell inserts (ThermoFisher). MDMs were stimulated with ARDS patients BALF from the HARP2 clinical trial for 24 hrs (ISRCTN88244364) before lysis in the kit lysis buffer containing protease and phosphatase inhibitors. The detection of phosphorylated proteins was visualized using chemiluminescent detection and the relative levels of protein phosphorylation (pixel density in each spot of the array minus background) was assessed between the samples using densitometry.

**Small RNA Next Generation Sequencing**

MSCs were stimulated with BALF (30% Cf) from ARDS patients for 24 hrs, EVs isolated and RNA including small RNAs extracted (miRNeasy, Qiagen). RNA integrity was assessed on Qubit™ RNA HS Assay Kit (Invitrogen). Small RNAs were converted to cDNA libraries using the NEXTFLEX® Small RNA-Seq Kit v3 (Perkin-Elmer). QC of the libraries and NGS was performed by the genomics core facility at Queens University Belfast on a NextSeq 550 System (Illumina). FASTq files were uploaded onto CLC Genomics Workbench using the small RNA pipeline/analysis tool (Qiagen Digital Insights). This tool was used for trimming of sequencing reads, counting and annotating the results using miRBase v21.

**Knockdown of SOCS1 expression and pharmaceutical targeting of STAT5 in MDMs**

Small interfering RNA (siRNA) transfection was used to knock down expression of SOCS1 in human MDMs. siRNAs included ON-TARGET *plus* Human SOCS1 SMARTPool siRNA and ON-TARGET plus non-targeting control Pool (Dharmacon). MDMs were seeded at 300,000

cells per well in 24-well plates and transfected with DharmaFECT Duo transfection reagent (Dharmacon). Briefly, siRNA (5 nmol) was diluted in 250 µl 1X siRNA buffer (5X siRNA buffer diluted with molecular grade RNase-free water, Dharmacon) to generate a 20 µM stock. A 5 µM siRNA solution was then prepared in 1X siRNA buffer. In separate tubes, the siRNA Tube 1 (2.5 µl 5 µM siRNA plus 47.5ul serum-free RPMI1640) and the DharmaFECT transfection reagent Tube 2 (1 µl plus 49ul serum-free RPMI1640) were prepared and left for 5 min at RT. These were then mixed together before incubation for a further 20min at RT. In each well, 100 µl of transfection complexes were added to 400 µl of 10% FBS antibiotic-free RPMI1640. The final concentration of siRNA was 25 nM. Cells were incubated at 37°C in 5% $CO_2$ for 48 hr. Western Blot was used to assess the transfection efficacy.

AC-4-130 (AOB36422, AOBIOUS), a specific STAT5 inhibitor that targets the SH2 domain of STAT5 was used to block STAT5 signalling in macrophages. 1mg AC-4-130 was dissolved in dimethyl sulfoxide (DMSO) to generate 1000 µM stock. 5 µM AC-4-130 or the same volume of DMSO in serum-free RPMI1640 was used to treat MDMs for 24 hrs before assessment using western blot.

**Overexpression of miRNA181a-5p in MDMs and MSCs**

Cells were transfected with miRIDIAN microRNA human miR-181a-5p mimic (Dy547-labeled, Dharmacon) or miRIDIAN microRNA mimic negative control (Dharmacon). MDMs were seeded at 300,000 cells per well in 24-well plates or MSCs at 200,000 cells per well in 6-well plates. The following day the cells were washed and media replaced with 200 µl Opti-MEM (ThemoFisher). Transfection complexes were generated in separate tubes and left at room temperature for 5 minutes. Tube 1 (2 µl Oligofectamine (ThermoFisher) was added to 7.5 µl Opti-MEM for MDMs, or 6 µl Oligofectamine was added to 22.5 µl Opti-MEM for MSCs) and for Tube 2 (1.25 µl 20 µM miR-181a-5p / miRNA scramble (negative control) was added to 40 µl Opti-MEM for MDMs, or 3.75 µl 20 µM miR-181a-5p / miRNA scramble (negative control) was added to 120 µl Opti-MEM for MSCs). Tubes 1 and 2 were mixed and left for 20min at RT, subsequently, 50 µl of transfection complexes were added to appropriate wells. After a 4 hr incubation, 125 µl Opti-MEM with 30% FBS was added to each well for 48 hr. Fluorescence imaging of live MDMs or MSCs was performed using the EVOS FL Auto Imaging System (Life technologies) at 10X magnification usually at 24 hrs after transfection.

**Transfection of MSCs with Locked nucleic acid (LNA) miR181a-5p**

HSA-miR181a-5p miRCURY locked nucleic acid (LNA) miRNA inhibitor (sequence 5'-ACTCACCGACAGCGTTGAATG - 3', QIAGEN) was transfected into MSCs to suppress

miR181a expression. A scrambled miRCURY LNA miRNA inhibitor (5'-TAACACGTCTATACGCCCA-3', QIAGEN) was included as a control (LNA miRNAscr-MSC-EVs). 5 nmol LNA miR181a-5p or scrambled LNA inhibitor was diluted in 250 µL nuclease free water to generate a 20 µM stock. MSCs were seeded at 200,000 cells per well in a 6 well plate. The following day, the cells were washed and media replaced with 600 µL Opti-MEM (ThemoFisher). Transfection complexes were generated in separate tubes and left at room temperature for 5 minutes. Tube 1 (6 µl Oligofectamine (ThermoFisher) was added to 22.5 µL Opti-MEM) and for Tube 2 (3.75 µL 20 µM miR-181a-5p LNA / miRNAscr LNA (negative control) was added to 120 µL Opti-MEM). Tubes 1 and 2 were mixed and left for 20 min at RT, subsequently 150 µL of transfection complexes was added to appropriate wells. After a 4 hr incubation, 375 µl Opti-MEM with 30% FBS was added to each well until 24 hrs after transfection when media was changed to serum free media for a further 48 hrs before EV extraction. MSC-EVs with miR181a LNA (LNA miR181a-MSC-EVs) or control i.e containing miR181a (LNA miRNAscr-MSC-EVs ) were extracted as outlined above (5).

### ***In Vivo*** **LPS-induced Acute Lung Injury Model**

Anesthetised C57BL/6 male mice (8- to 10-week-old; Harlan Laboratories Ltd, UK) were administered LPS *E. Coli* LPS O111:B4 (Millipore, #3526781) in saline at 2 mg/kg of body weight intratracheally, facilitated by a laryngoscope. Four hours after the instillation, mice were divided into groups and given nothing (untreated control), PBS or MSC-EVs (MSC EVs untreated, LNA miRNA181-MSC-EVs or LNA miRNAscr-MSC-EVs) via intravenous injection of the lateral caudal vein. 24 hrs after LPS administration, mice were euthanized and broncho-alveolar lavage fluid (BALF) extracted and processed for alveolar macrophage (AMs) isolation. BALF was centrifuged at 400 x *g* at 4°C for 10 minutes and the supernatant was collected for testing. The cell pellet was resuspended in 200µL eBioscience™ 1X RBC lysis buffer (Invitrogen, ThermoFisher) and incubated at room temperature for 3-4 minutes with occasional shaking, before centrifugation at 400 x *g* at 4°C for 5 minutes. Cells were then resuspended in 200 µL DPBS (Gibco). The total number of leukocytes was estimated in each sample using trypan blue exclusion assay.

### **Cytospin Preparation**

BALF cells were centrifuged onto Polysine microscope slides (ThemoFisher) at 3,000 RPM for 5 minutes using the StatSpin Cytofuge2 (Beckman Coulter, VWR). The cytoslides were dried overnight before staining with the Speedy Diff Complete kit (Clin-Tech). Cells were visualised in brightfield using a Leica DM5500 microscope (Leica) at 20X magnification and the number of neutrophils estimated in ≥400 BALF cells using imageJ.

## Immunofluorescence Staining

BALF cells were resuspended in 200 µL Dulbecco's Modified Eagle Medium/F12 medium supplemented with 1% Penicillin-Streptomycin and 10% FBS (DMEM/F12-10) and seeded in CC2 glass chamber (100 µL per well) (mimics polylysine, Nunc$^{TM}$ Lab-Tek$^{TM}$ II Chamber Slide system, ThermoFisher) overnight. Slides were then washed in DPBS and fixed using 100uL fixative solution (Image-iT$^{TM}$ Fixative Solution, 4% formaldehyde, methanol-free, ThermoFisher) for 1 hr at RT. Cells were permeabilised using 100 µL ice-cold 100% methanol at -20°C for 10 min and blocked using 10% normal donkey serum at RT for 2 hrs. AMs were then incubated with primary antibodies diluted in 1% BSA in PBS-T (PBS with 0.3% Tween100) at 1:200 dilution overnight at 4°C. Primary antibodies included; CD68 Mouse mAb #FA-11 (ThermoFisher), Phospho-Stat5 (Tyr694, C71E5) Antibody (Cell Signalling Technology) and SOCS1 (ab9870) Antibody (Abcam). The next day AMs were washed using PBS and incubated with secondary antibodies diluted in 1% BSA in TBS-T (TBS with 0.3% Tween100) at a 1:500 dilution for 1h at RT. Primary/secondary antibody combinations were as follows; pSTAT5-Alexa 488 #4412S (Invitrogen); CD68-Alexa 555 #ab150114(Abcam); SOCS1-Alexa 647 #ab150135(Abcam). DAPI (300nM, Invitrogen) was used to stain nuclei and slides were then mounted using Vectashield HardSet Antifade Mounting Medium (H-1400-10, Vectorlabs). An SP8 inverted confocal microscope (Leica, TCS SP8, Germany) was used to image the fluorescence at 100X magnification using the LAS X software.

## Statistical analysis

Statistical analyses were performed using Prism 7 software (GraphPad, USA). Experiments for MDMs were performed using at least three different donors, the average of three technical replicates was taken as a single data point for each donor, and the data points were pooled together for statistical analysis. Pooled data were scored for normality by Shapiro-Wilk test and presented as the mean with SD. For parametric data, two-tailed Student's t-test or one-way ANOVA followed by Bonferroni post hoc test was used. For non-parametric data, Mann-Whitney U test or one-way ANOVA followed by Dunn's selected comparisons was used. Statistical significance is represented as *$p < 0.05$ or **$p < 0.01$.

## Reference:

1. Dominici M, Le Blanc K, Mueller I, Slaper-Cortenbach I, Marini FC, Krause DS, et al. Minimal criteria for defining multipotent mesenchymal stromal cells. The International Society for Cellular Therapy position statement. Cytotherapy. 2006;8(4):315–7.

2. Théry C, Witwer KW, Aikawa E, Alcaraz MJ, Anderson JD, Andriantsitohaina R, et al.

Minimal information for studies of extracellular vesicles 2018 (MISEV2018): a position statement of the International Society for Extracellular Vesicles and update of the MISEV2014 guidelines. J Extracell Vesicles. 2018;7(1).

3. Silva JD, Su Y, Calfee CS, Delucchi KL, Weiss D, McAuley DF, et al. MSC extracellular vesicles rescue mitochondrial dysfunction and improve barrier integrity in clinically relevant models of ARDS. Eur Respir J. 2020;57(6):2002978.

4. Reilly JP, Calfee CS, Christie JD. Acute Respiratory Distress Syndrome Phenotypes. Vol. 40, Seminars in Respiratory and Critical Care Medicine. 2019. p. 19–30.

5. Ørom UA, Kauppinen S, Lund AH. LNA-modified oligonucleotides mediate specific inhibition of microRNA function. Gene. 2006;
