# Supplementary material for: Mesenchymal stromal cells-derived extracellular vesicles reprogramme macrophages in ARDS models through the miR-181a-5p-PTEN-pSTAT5-SOCS1 axis
Source: Thorax. 2022 Aug 10;78(6):617–30. doi: 10.1136/thoraxjnl-2021-218194 (PMC10314068; doi:10.1136/thoraxjnl-2021-218194)
Supplement: Supplementary data [file thoraxjnl-2021-218194supp002.pdf]

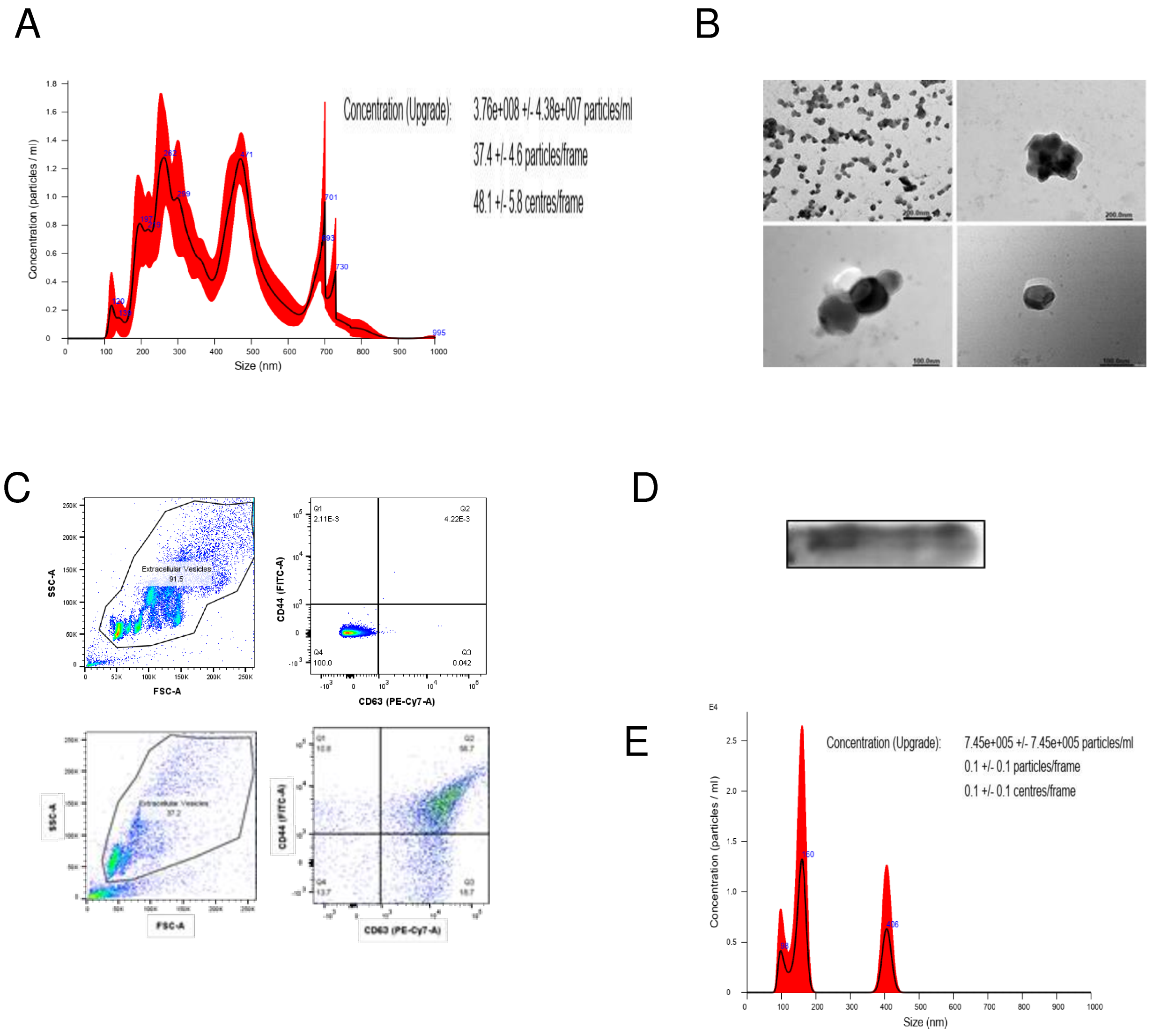

Supplementary Figure 1. Characterization of MSC-derived extracellular vesicles (MSC-EVs). **A,** Nanoparticle tracking analysis of MSC-EVs. **B,** Representative Transmission Electron Microscopy images taken using TEM microscope (JEOL, JEM 1400Plus, Japan) showing extracellular vesicles morphology (upper images, scale bar 200nm; bottom images, scale bar 100nm). **C,** Representative flow cytometry plots of MSC-EVs conjugated with 4µm beads demonstrating presence of EV populations unstained (upper panels) and double positive (bottom panels) for CD44 and CD63 (56.7%). **D,** Immunoblot for protein expression levels of CD63 in MSC-EVs lysates. **E,** Nanoparticle tracking analysis of MSC CM supernatants after EV isolation.
